# Collaboration Between Maternal and Child Health and Chronic Disease Epidemiologists to Identify Strategies to Reduce Hypertension-Related Severe Maternal Morbidity

**DOI:** 10.5888/pcd16.190045

**Published:** 2019-12-12

**Authors:** Ghasi Phillips-Bell, Abigail Holicky, Megan Macdonald, Leticia Hernandez, Angel Watson, Rahel Dawit

**Affiliations:** 1 Division of Community Health Promotion, Florida Department of Health, Tallahassee, Florida; 2 Division of Reproductive Health, Centers for Disease Control and Prevention, Atlanta, Georgia; 3 Centers for Disease Control and Prevention, Council of State and Territorial Epidemiologists Applied Epidemiology Fellowship, Atlanta, Georgia

## Abstract

**Introduction:**

Maternal and child health (MCH) and chronic disease programs at state health agencies may not routinely collaborate. The objective of this study was to describe a project that enhanced relationships between MCH and chronic disease epidemiologists at the Florida Department of Health, increased epidemiologic capacity, and informed both programs.

**Methods:**

We collaborated to assess hypertension-related severe maternal morbidity (H-SMM) and hypertensive disorders (preexisting hypertension, gestational hypertension, and preeclampsia) among women at delivery of their live birth to help determine the burden on health care systems in Florida. We identified ways to improve the health of women before they conceive and to help them manage any chronic diseases during the perinatal period.

**Results:**

We found differences by maternal characteristics in H-SMM rates among 979,660 women who delivered live births. We proposed strategies to support collaboration between state MCH and chronic disease staff. First, increase the screening, monitoring, and management of hypertension before, during, and after pregnancy. Second, examine H-SMM concurrently with maternal mortality to help find prevention strategies. Third, include reproductive-aged women in ongoing hypertension prevention and intervention efforts. Fourth, expand team-based care to include obstetricians, midwives, and doulas who can work together with primary care providers for hypertension management. And fifth, create and share data products that guide various groups about hypertension and related risk factors among reproductive-aged women.

**Conclusion:**

The collaboration between the Florida Department of Health MCH and chronic disease epidemiologists produced 1) a program-relevant indicator, H-SMM and 2) strategies for enhancing program and clinical activities, communication, and surveillance to reduce H-SMM rates.

**Summary**

**What is already known on this topic?**

Maternal and child health (MCH) outcomes and chronic diseases can be interconnected. At the Florida Department of Health, MCH programs examine how hypertensive disorders affect maternal morbidity and mortality, and chronic disease programs focus on how hypertension affects the general population of men and women.

**What is added by this report?**

We described an example of MCH and chronic disease staff members collaborating to develop a meaningful program indicator — hypertension-related severe maternal morbidity — and potential data-to-action strategies to integrate efforts.

**What are the implications for public health practice?**

The relationship between MCH and chronic disease program staff members can be enhanced by intertwining efforts, unique perspectives, and resources on projects with shared visions and objectives.

## Introduction

Maternal and child health (MCH) and chronic disease epidemiologists may not routinely work together at state health agencies. Possible reasons include organizational structures that preclude effective communication and a limited understanding of how MCH and chronic disease morbidity intersect (1). For example, women with chronic conditions, such as diabetes or obesity, have an increased risk of preeclampsia (2), a pregnancy-specific hypertensive disease that involves several body systems and typically starts after the 20th week of pregnancy (3). Additionally, pregnancy can unmask chronic diseases or a propensity for chronic diseases after pregnancy and later in life (4). Furthermore, chronic diseases have been cited as contributing factors to rising national and state-level maternal mortality rates (5–7). Effectively addressing topics that cross both disciplines, such as severe maternal morbidity (SMM), requires expertise in both MCH and chronic disease.

SMM refers to unexpected outcomes of labor and delivery that result in major consequences to women’s health (8). SMM increases medical costs, lengthens hospitalization stays (8), and increases the risk of postpartum maternal morbidity and mortality, particularly among pregnant women with hypertensive disorders (9). SMM rates have been increasing nationally (8), thereby warranting state-level examination, which could help improve maternal health by promoting better health services and supporting the development of effective health policies at state and national levels (10). Our team of MCH and chronic disease epidemiologists at the Florida Department of Health (FDOH) previously analyzed data on SMM and hypertensive disorders among delivery hospitalizations.

The objective of this study was to describe our collaborative process to further analyze SMM data, develop a program-relevant measure — hypertension-related SMM (H-SMM) — and identify opportunities for translating H-SMM results into potential public health practices.

## Methods

During 2014–2016, our team of MCH and chronic disease epidemiologists participated in a capacity-building program sponsored by the Centers for Disease Control and Prevention (CDC) and taught by staff at the University of Illinois at Chicago School of Public Health (11). Our participation was the impetus for collaborating to analyze claims-based data, such as hospital discharge and Medicaid data.

First, we analyzed SMM data and observed that Florida and national SMM rates (per 10,000 delivery hospitalizations) were similar: 162.4 in Florida during 2010–2013 (12) and 162.8 in the nation during 2010–2011 (8). We also found that the risk of SMM was more than twice as high among women with chronic hypertension or gestational hypertension than among women without these conditions in Florida (12).

We then shared those results with staff members who administered or supported various MCH or chronic disease programs to receive guidance on further analyses. To best meet Florida’s program needs, the staff advised the following:

- Hypertension is an important risk factor for other adverse MCH outcomes, including maternal mortality (13,14).
- Further analyses of SMM, specifically related to hypertension, could inform current MCH and chronic disease programs because the prevalence of hypertension is increasing among women of reproductive age (15).
- Measures of frequency or disease burden (eg, rates, length of stay, hospital charges) are more understandable and useful than measures of association (eg, risk ratios). Measures of frequency describe the effect of health outcomes on populations, making these data more useful and more likely than other types of data to resonate with public health practitioners, government leadership, and other nongovernmental organizations such as health care systems. In contrast, measures of association are useful for conducting more in-depth analyses, such as estimating causal associations with minimal confounding or other bias.

We used this information to focus on hypertensive disorders and SMM. Rates of SMM among delivery hospitalizations (16) can be calculated from state hospital discharge data using a 25-condition definition established by Callaghan and colleagues (17). The established 25-condition SMM definition included many conditions unrelated to hypertensive disorders. Consequently, we consulted with a senior clinical epidemiologist specializing in chronic disease to identify a subset of the established 25 conditions that could be pathologically related to hypertensive disorders (Table 1). We defined this subset as hypertension-related SMM (H-SMM). We identified H-SMM by using *International Classification of Diseases, Ninth Revision, Clinical Modification* (ICD-9-CM) codes in the hospital discharge data (18). As such, H-SMM was defined as having 1 or more of the following conditions: acute renal failure, cardiac arrest/ventricular fibrillation, heart failure during procedure or surgery, conversion of cardiac rhythm, acute myocardial infarction, pulmonary edema, disseminated intravascular coagulation, thrombotic embolism, puerperal cerebrovascular disorders, eclampsia, or aneurysm. We created the H-SMM binary indicator to categorize women who had at least 1 H-SMM condition or had none.

**Table 1:** ICD-9-CM Codes Used To Define Hypertension-Related Severe Maternal Morbidity and Hypertensive Disorders, Florida, 2010–2014

Condition(s)	ICD-9-CM Code(s)
**Hypertension-related severe maternal morbidity^a^**	
Acute renal failure	584.x, 669.3x
Cardiac arrest/ventricular fibrillation	427.41, 427.42, 427.5
Heart failure during procedure or surgery	669.4x, 997.1
Conversion of cardiac rhythm	99.6x
Acute myocardial infarction	410.xx
Pulmonary edema	428.1, 518.4
Disseminated intravascular coagulation	286.6, 286.9, 666.3x
Thrombotic embolism	415.1x, 673.0x, 673.2x, 673.3x, 673.8x
Puerperal cerebrovascular disorders	430, 431, 432.x, 433.xx, 434.xx, 436, 437.x, 671.5x, 674.0x, 997.2, 999.2
Eclampsia	642.6x
Aneurysm	441.xx
**Preexisting hypertension**	401.0, 401.1, 401.9, 402.00, 402.01, 402.10, 402.11, 402.90, 402.91, 403.00, 403.01, 403.10, 403.11, 403.90, 403.91, 405.01, 405.09, 405.11, 405.19, 405.91, 405.99, 459.30, 459.31, 459.32, 459.33, 459.39, 572.3, 642.01, 642.02, 642.11, 642.12, 642.21, 642.22, 642.90, 642.91, 642.92, 642.93, 642.94
**Gestational hypertension**	642.31, 642.32, 642.33 642.34
**Preeclampsia**	642.40, 642.41, 642.42, 642.43, 642.44, 642.50, 642.51, 642.52, 642.53, 642.54

Abbreviations: ICD-9-CM, *International Classification of Diseases, Ninth Revision, Clinical Modification*.

a Classification includes ≥1 listed condition.

We also examined H-SMM rates among pregnant women with the following subtypes of hypertensive disorders: 1) preeclampsia, 2) preexisting hypertension, and 3) gestational hypertension. We examined each subtype separately. We used a hierarchical approach to classify women into only 1 subtype when codes for multiple conditions were present. First, women with preeclampsia were identified, regardless of other hypertension-related codes, then those with preexisting hypertension, followed by those with gestational hypertension. These subtypes differ clinically and can have varying associations with adverse birth outcomes (9). The subtypes were identified by using ICD-9-CM codes in the hospital discharge data set (Table 1).

We analyzed hospital deliveries during 2010–2014 (N = 979,660) by selected maternal characteristics using Florida hospital discharge data linked to birth certificate data. We investigated median hospital length of stay, median hospital charges, and H-SMM rates, and whether these differed by type of hypertensive disorder, to help identify the burden each disorder places on health care systems in Florida. We tabulated prevalence ratios, but we did not interpret these data because they resulted from ad hoc analyses, not discussions with programmatic staff. The FDOH Institutional Review Board approved this analytic project.

## Results

Of 979,660 delivery hospitalizations during 2010–2014 in Florida, 4,021 recorded at least 1 H-SMM disorder — a rate of 41.0 cases per 10,000 delivery hospitalizations (Table 2). Among categories of race/ethnicity, the H-SMM rate was highest among women who were non-Hispanic black (65.0 cases per 10,000 delivery hospitalizations); among categories of pre-pregnancy body mass index, the rate was highest among women who had obesity (50.3 cases per 10,000 delivery hospitalizations), and among categories of hypertensive disorders, the rate was highest among women who had preeclampsia (198.9 per 10,000 delivery hospitalizations).

**Table 2:** Frequencies, Rates, and Unadjusted Prevalence Ratios for Overall and Hypertension-Related Severe Maternal Morbidity Among Delivery Hospitalizations, by Maternal Characteristics, Florida, 2010–2014

Maternal Characteristic	All Delivery Hospitalizations, N^a^	No. of Delivery Hospitalizations With H-SMM^a^^,^ b	H-SMM Rate Per 10,000 Delivery Hospitalizations	Unadjusted Prevalence Ratio (95% CI)	*P* Value^c^
**Overall**	979,660	4,021	41.0	NA	NA
**Race/ethnicity**					
Non-Hispanic white	443,373	1,392	31.4	1.0 [Reference]	<.001
Non-Hispanic black	223,159	1,451	65.0	2.1 (1.9–2.2)	
Hispanic	258,615	953	36.9	1.2 (1.1–1.3)	
Non-Hispanic other	48,526	196	40.4	1.3 (1.1–1.5)	
**Pre-pregnancy body mass index (kg/m^2^)**					
Underweight (<20.0)	43,246	133	30.8	0.9 (0.7–1.1)	<.001
Normal weight (20.0–24.9)	440,716	1,534	34.8	1.0 [Reference]	
Overweight (25.0–29.9)	233,272	945	40.5	1.2 (1.0–1.3)	
Obese (≥30.0)	207,224	1,044	50.3	1.4 (1.3–1.6)	
**Hypertensive disorders**					
No hypertension	873,074	2,614	29.9	1.0 [Reference]	<.001
Preexisting hypertension	28,600	391	136.7	4.5 (4.1–5.1)	
Gestational hypertension	37,507	211	56.3	1.9 (1.6–7.2)	
Preeclampsia	40,479	805	198.9	6.6 (6.1–7.2)	

Abbreviations: CI, confidence interval; H-SMM, hypertension-related severe maternal morbidity.

a Values in some categories do not sum to the value in the column header because of missing data.

b H-SMM is defined as having 1 or more of the following conditions: acute renal failure, cardiac arrest/ventricular fibrillation, heart failure during procedure or surgery, conversion of cardiac rhythm, acute myocardial infarction, pulmonary edema, disseminated intravascular coagulation, thrombotic embolism, puerperal cerebrovascular disorders, eclampsia, or aneurysm.

c Calculated by using the Kruskal–Wallis equality-of-populations rank test.

Women with H-SMM used more health services — resulting in higher median length of stay and hospital charges — than women with no H-SMM. Women with preexisting hypertension or preeclampsia used more health services than those with gestational hypertension or those without hypertension. Lastly, we observed a greater burden on the health care system when H-SMM and hypertensive disorders were experienced simultaneously. Women with H-SMM and either preexisting hypertension or preeclampsia had a significantly longer length of stay (2 days longer) and higher hospital charges (approximately $18,000 more) than those without H-SMM with the same hypertensive disorders.

### Recommended data-to-action strategies

Given these findings, our team proposed the following strategies that our state health agency and its partners could use to support MCH and chronic disease efforts.

#### Strategy 1: Increase screening, monitoring, and guideline-based management of hypertension among women of reproductive age before, during, and after pregnancy

In our study, women with preexisting hypertension and preeclampsia incurred higher charges and longer hospital stays. This finding suggests that standardized, evidence-based interventions to prevent and manage hypertensive disorders during the preconception and prenatal periods could be critical for reducing morbidity and mortality (19).

The American Heart Association added preeclampsia to its list of risk factors for later-life cardiovascular disease. They recommended adding preeclampsia to the method for calculating the risk score for cardiovascular disease and posited that cardiovascular disease should be tested before the age of 40 years among women with a history of preeclampsia (3).

#### Strategy 2: Examine H-SMM and SMM concurrently with maternal mortality to help identify upstream prevention strategies

Our data indicate H-SMM is more prevalent among women with hypertension than among women without hypertension. Given that women with hypertension also have increased risk of pregnancy-related mortality, statewide programs throughout the nation, including perinatal quality collaboratives (groups that work to improve perinatal health care, quality, and patient safety) (20) and maternal mortality review committees (stakeholders who identify and review maternal deaths and develop prevention recommendations), could examine H-SMM and incorporate this indicator into their work. Trends in both SMM and H-SMM should be considered together with trends and characteristics of maternal mortality to find prevention strategies that work earlier on the continuum of maternal health. The Florida Pregnancy-Associated Mortality Review Committee identified hypertensive disorders as major risk factors for pregnancy-related deaths in the state. Definitions of pregnancy-related deaths and pregnancy-associated deaths are not standardized across the nation. Florida defines the former as deaths due to factors related to the actual pregnancy (eg, pregnancy-induced hypertension) and the latter as deaths that include pregnancy-related deaths plus deaths unrelated to the pregnancy (eg, vehicular accidents).

CDC has offered 5-year funding support for maternal mortality review committees to identify and characterize maternal mortality to better understand pregnancy-related deaths, develop recommendations for prevention, improve quality of care, and address social determinants of health to reduce health inequities. Grantees who seek to expand their work may find Strategy 2 useful for identifying prevention factors. Additionally, federal partners might consider funding opportunities for programs looking to integrate SMM and maternal mortality surveillance efforts.

#### Strategy 3: Include women of reproductive age in ongoing hypertension prevention and intervention efforts

FDOH’s Bureau of Chronic Disease Prevention (BCDP) works to optimize the use of electronic health records in health systems, including county health departments and federally qualified health centers. BCDP also alerts health professionals about which patients may have undiagnosed hypertension and may require monitoring. These interventions include algorithms specific to certain blood pressure readings and exclude pregnant women. In our study, women with preexisting hypertension had higher H-SMM rates, longer lengths of stay, and greater hospital charges than those without hypertension. Our findings support enhancing the next phase of the electronic health record intervention to include women of reproductive age by pregnancy status into the algorithm, which will ensure the timely diagnosis of hypertension and help prevent pregnancy-related disease and death.

BCDP also works with the FDOH-funded Medication Therapy Management Support project, which trains community health workers (CHWs) to work in health care systems to help patients better manage their hypertension. CHWs primarily work in low-income communities with limited access to health care and in populations that face multiple barriers to optimal health outcomes, including non-Hispanic black women. We found that H-SMM was more prevalent among non-Hispanic black women than among members of other racial/ethnic groups, a finding that was similar to findings in another report (21). We suggest enhancing the CHW program to reach women of reproductive age with preexisting hypertension to ensure they receive proper care to improve their health and lower their risk of pregnancy-related morbidity and mortality. CHWs could also be trained to pay close attention to pregnant women with preeclampsia to further expand the scope of the project.

BCDP plans to work with health care systems in Florida to help them become recognized as Hypertension Control Champions through the Million Hearts Initiative (22). This initiative recognizes health care systems that achieve blood pressure control for at least 70% of their patients with hypertension by using evidence-based programs. Uncontrolled preexisting hypertension is a risk factor for preeclampsia, which can lead to cardiovascular diseases (23). Adequate management of preexisting hypertension before and during pregnancy may reduce the burden of preeclampsia on health care systems. In our study, women with preexisting hypertension and preeclampsia had the highest median length of stay and hospital charges. MCH and chronic disease prevention programs could collaborate to help providers who include women of reproductive age in their efforts to become Hypertension Control Champions.

#### Strategy 4: Expand team-based care to include obstetricians, midwives, and doulas who collaborate with primary care providers for hypertension management

BCDP works with partners to implement team-based care. This evidence-based approach uses nonphysician and physician provider care teams to help patients better manage their chronic conditions. Women in our sample with preexisting hypertension had an H-SMM rate 3 times higher than the overall rate and used health care services at a higher rate. We suggest expanding primary care teams to include obstetricians, midwives, and doulas for bidirectional consultation for women of reproductive age. Primary care providers can encourage their chronic disease patients seeking pregnancy to obtain a preconception consultation with an obstetric care provider and conversely obstetric providers can assist postpartum patients in following up with their primary care physicians to appropriately manage hypertension after pregnancy.

An example of such an expansion is North Carolina’s Pregnancy Care Management Program, which pairs low-income women who have medical needs with a social worker or nurse care manager to help address factors that affect low birth weight and preterm birth (24). The care managers communicate with providers to support the medical plan, inform providers about social conditions (eg, lack of stable housing, food insufficiency, intimate partner violence) that may jeopardize care and work to help resolve these social conditions. Although this program aims to reduce adverse birth outcomes, the model can be applied to maternal morbidity to manage complications during the prenatal period and ensure proper follow-up for hypertensive disorders.

#### Strategy 5: Create and share data products to guide the public, health care providers, and partner agencies about hypertension and related risk factors among reproductive-aged women

BCDP is creating a communications plan to identify, produce, and share data products, such as infographics and social media posts, to inform the public, health care providers, and partners about hypertension and related risk factors. MCH and chronic disease prevention programs could collaborate to adapt these products for women of reproductive age, especially women with preexisting hypertension who are at increased risk of H-SMM during pregnancy. Communication products for the general public should adhere to key components of effective health communication and patient education (eg, use plain, nonmedical language; organize information into 2 or 3 components) (3). Materials developed should be shared at the same time with partners in both fields and posted on both MCH and chronic disease prevention program websites.

## Discussion

We described an example of positive work that can emerge from partnerships between MCH and chronic disease staff members at state health agencies. In Florida, this collaboration led to the development and analysis of an H-SMM indicator that can be incorporated into surveillance, monitoring, and evaluation activities. Similarly, health agencies in Ohio, Missouri, and West Virginia implemented collaborative efforts between MCH and chronic disease programs to improve the health outcomes of women at risk for type 2 diabetes (25). This model of collaboration could be replicated in other state health departments that are working to reduce chronic health conditions that affect maternal morbidity and mortality.

A strength of our study was the ability to collect valuable feedback and obtain support from program administrators, clinicians, and agency leaders with the authority to implement relevant interventions. In addition, our study also contributes to the body of research on SMM and other adverse pregnancy-related hypertension outcomes (26,27). We identified a subset of the overall 25-condition SMM index relevant to MCH and chronic disease prevention efforts to create the H-SMM indicator. The results obtained from analyzing this indicator could be used to enhance chronic disease management protocols by expanding existing services to pregnant and postpartum women with hypertensive disorders, especially to non-Hispanic black women or women who have obesity before pregnancy. This expansion could also help to ensure that providers who regularly see reproductive-aged women (eg, obstetricians/gynecologists) are helping to properly diagnose and manage these conditions. They can also use patient encounters to bolster the development and use of reproductive life plans, which are tools to help patients achieve their personal health goals in conjunction with pregnancy timing and occurrence.

Our work had challenges. The level of collaboration required by our work was not routine or established at FDOH; thus, it called for a redistribution of current workload and allocation of time for team meetings. We were fortunate to have supportive management who approved staff participation in this capacity-building effort and that an underlying goal of this project (ie, examine hypertension-related maternal morbidity to help improve women’s health) has been identified as a priority in Florida’s State Health Improvement Plan (28). Strong leadership within state projects is essential for prioritizing progress and facilitating collaboration. To build capacity for implementing collaborative projects, state health agencies should consider local workforce development opportunities, such as internships provided by nearby universities. They may also consider applying for host interns or fellows from various national organizations, including, but not limited to, CDC and the Council for State and Territorial Epidemiologists (29).

Our analysis has several limitations. It was subject to limitations of the ICD-9-CM codes described elsewhere (10), including that the hypertensive disorders used to create the H-SMM indicators could be based on incorrect ICD-9-CM codes. In addition, the case definition of H-SMM was sensitive, but it might not be specific to hypertension because we included conditions that could be associated with hypertension (eg, pulmonary edema) (30). This approach allowed us to capture data on a larger number of cases possibly related to hypertension, but it limited our ability to determine whether cases were true hypertensive disorders. Furthermore, since the completion of our analysis, the definition of SMM was revised to include 21 conditions and the codes were updated to reflect the transition from ICD-9-CM to ICD-10-CM (8). Thus, our results may not be comparable to SMM analyses that use administrative hospital discharge data from October 2015 forward, but they could be adapted for use with current coding classifications.

This collaborative experience enhanced our understanding of claims-based analysis and enriched our professional relationships. Although external skill-building activities can help agencies initiate multidisciplinary partnerships, MCH and chronic disease epidemiologists can also benefit from examining their existing infrastructure to find ways to develop and sustain collaborations on program-relevant projects. No matter the size, state health agencies by design have multidisciplinary capacity and thus are in unique positions to serve as best practice examples for fostering collaboration and aligning activities for maximum effect. Actively strengthening these relationships by intertwining efforts, unique perspectives, and resources on projects with shared objectives may ultimately benefit and guide the enhancement of MCH and chronic disease programs.

## References

[1] Collins JL , Lehnherr J , Posner SF , Toomey KE . Ties that bind: maternal and child health and chronic disease prevention at the Centers for Disease Control and Prevention. Prev Chronic Dis 2009;6(1):A01. 19080007 PMC2644605

[2] Persson M , Cnattingius S , Wikström AK , Johansson S . Maternal overweight and obesity and risk of pre-eclampsia in women with type 1 diabetes or type 2 diabetes. Diabetologia 2016;59(10):2099–105. 10.1007/s00125-016-4035-z 27369871 PMC5016540

[3] American College of Obstetricians and Gynecologists; Task Force on Hypertension in Pregnancy. Hypertension in pregnancy. Report of the American College of Obstetricians and Gynecologists’ Task Force on Hypertension in Pregnancy. Obstet Gynecol 2013; 122(5):1122–31. 24150027 10.1097/01.AOG.0000437382.03963.88

[4] Mosca L , Benjamin EJ , Berra K , Bezanson JL , Dolor RJ , Lloyd-Jones DM , Effectiveness-based guidelines for the prevention of cardiovascular disease in women — 2011 update: a guideline from the American Heart Association. Circulation 2011;123(11):1243–62. 10.1161/CIR.0b013e31820faaf8 21325087 PMC3182143

[5] Nelson DB , Moniz MH , Davis MM . Population-level factors associated with maternal mortality in the United States, 1997–2012. BMC Public Health 2018;18(1):1007. 10.1186/s12889-018-5935-2 30103716 PMC6090644

[6] Building US Capacity to Review and Prevent Maternal Deaths. Report from nine maternal mortality review committees. 2018. http://reviewtoaction.org/Report_from_Nine_MMRCs. Accessed January 17, 2019.

[7] Florida Department of Health. Florida’s pregnancy-associated mortality review: 2016 update. 2018. http://www.floridahealth.gov/statistics-and-data/PAMR/_documents/pamr-2016-update.pdf. Accessed January 17, 2019.

[8] Centers for Disease Control and Prevention. Severe Maternal Morbidity in the United States. 2017. http://www.cdc.gov/reproductivehealth/MaternalInfantHealth/SevereMaternalMorbidity.html. Accessed January 15, 2019.

[9] Gongora MC , Wenger NK . Cardiovascular complications of pregnancy. Int J Mol Sci 2015;16(10):23905–28. 10.3390/ijms161023905 26473833 PMC4632731

[10] Callaghan WM , Mackay AP , Berg CJ . Identification of severe maternal morbidity during delivery hospitalizations, United States, 1991–2003. Am J Obstet Gynecol 2008;199(2):133.e1–8. 10.1016/j.ajog.2007.12.020 18279820

[11] Phillips G , Sappenfield W , Handler A , Kogan MD . Promoting a trained MCH epidemiology workforce in state public health agencies through strategies developed from continued partnerships. Matern Child Health J 2012;16(Suppl 2):376–80. 10.1007/s10995-012-1095-7 22961362 PMC4539271

[12] Holicky A , Reid K , Thompson D , Phillips-Bell G , Hernandez L , Watson A , Severe maternal morbidity in Florida, 2010–2014. Proceedings of the Council of State and Territorial Epidemiologists Conference, Maternal and Child Health Symposium; 2015 Jun 15; Boston, MA.

[13] Berg CJ , Callaghan WM , Syverson C , Henderson Z . Pregnancy-related mortality in the United States, 1998 to 2005. Obstet Gynecol 2010;116(6):1302–9. 10.1097/AOG.0b013e3181fdfb11 21099595

[14] Campbell KH , Savitz D , Werner EF , Pettker CM , Goffman D , Chazotte C , Maternal morbidity and risk of death at delivery hospitalization. Obstet Gynecol 2013;122(3):627–33. 10.1097/AOG.0b013e3182a06f4e 23921870

[15] National Center for Health Statistics. Health, United States, 2015: with special feature on racial and ethnic health disparities, 2016. www.cdc.gov/nchs/data/hus/hus15.pdf. Accessed August 31, 2016. 27308685

[16] Kuklina EV , Whiteman MK , Hillis SD , Jamieson DJ , Meikle SF , Posner SF , An enhanced method for identifying obstetric deliveries: implications for estimating maternal morbidity. Matern Child Health J 2008;12(4):469–77. 10.1007/s10995-007-0256-6 17690963

[17] Callaghan WM , Creanga AA , Kuklina EV . Severe maternal morbidity among delivery and postpartum hospitalizations in the United States. Obstet Gynecol 2012;120(5):1029–36. 10.1097/AOG.0b013e31826d60c5 23090519

[18] Council on Patient Safety in Women’s Health Care. AIM SMM codes list 2015. http://safehealthcareforeverywoman.org/aim-program/aim-data/. Accessed July 28, 2017.

[19] American College of Obstetricians and Gynecologists . ACOG Committee opinion no. 767: emergent therapy for acute-onset, severe hypertension during pregnancy and the postpartum period. Obstet Gynecol 2019;133(2):e174–80. 10.1097/AOG.0000000000003075 30575639

[20] University of South Florida, Florida Perinatal Quality Collaborative. Hypertension in Pregnancy. 2017. http://health.usf.edu/publichealth/chiles/fpqc/hip. Accessed July 28, 2017.

[21] Creanga AA , Bateman BT , Kuklina EV , Callaghan WM . Racial and ethnic disparities in severe maternal morbidity: a multistate analysis, 2008-2010. Am J Obstet Gynecol 2014;210(5):435.e1–8. 10.1016/j.ajog.2013.11.039 24295922

[22] Centers for Disease Control and Prevention. Million Hearts Hypertension Control Champions. 2016. http://millionhearts.hhs.gov/partners-progress/champions/index.html. Accessed December 13, 2016.

[23] Leeman L , Dresang LT , Fontaine P . Hypertensive disorders of pregnancy. Am Fam Physician 2016;93(2):121–7. 26926408

[24] Kansagra SM , Herndon S , Kimple KS , Thomas C , Tomlinson S , Moore Z , Redefining the team in team-based care: role of public health. N C Med J 2018;79(4):235–9. 10.18043/ncm.79.4.235 29991615

[25] Association of Maternal & Child Health Programs. MCH and chronic disease collaboration. http://www.amchp.org/programsandtopics/womens-health/projects/chronic-disease/Pages/default.aspx. Accessed April 30, 2019.

[26] Potti S , Jain NJ , Mastrogiannis DS , Dandolu V . Obstetric outcomes in pregnant women with diabetes versus hypertensive disorders versus both. J Matern Fetal Neonatal Med 2012;25(4):385–8. 10.3109/14767058.2011.580403 21627547

[27] Zhang J , Meikle S , Trumble A . Severe maternal morbidity associated with hypertensive disorders in pregnancy in the United States. Hypertens Pregnancy 2003;22(2):203–12. 10.1081/PRG-120021066 12909005

[28] Florida Department of Health. Florida 2017–2021 state health improvement plan. http://www.floridahealth.gov/about/state-and-community-health-assessment/index.html. Accessed April 30, 2019.

[29] Centers for Disease Control and Prevention. Fellowships, internships, and learning opportunities. https://www.cdc.gov/fellowships/index.html. Accessed April 15, 2019.

[30] Murray JF . Pulmonary edema: pathophysiology and diagnosis. Int J Tuberc Lung Dis 2011;15(2):155–60, i. 21219673

